# Identification of CRISPR and riboswitch related RNAs among novel noncoding RNAs of the euryarchaeon *Pyrococcus abyssi*

**DOI:** 10.1186/1471-2164-12-312

**Published:** 2011-06-13

**Authors:** Kounthéa Phok, Annick Moisan, Dana Rinaldi, Nicolas Brucato, Agamemnon J Carpousis, Christine Gaspin, Béatrice Clouet-d'Orval

**Affiliations:** 1 Laboratoire de Microbiologie et Génétique Moléculaire, UMR 5100, Centre National de la Recherche Scientifique et Université de Toulouse III, 31062 Toulouse, France; 2 Laboratoire d'Intelligence artificielle, UR 875-INRA, 31326 Auzeville-Tolosan, France; 3 Laboratoire d'Anthropobiologie Moléculaire et Imagerie de Synthèse, UMR 5288, Centre National de la Recherche Scientifique, 31073 Toulouse, France

## Abstract

**Background:**

Noncoding RNA (ncRNA) has been recognized as an important regulator of gene expression networks in Bacteria and Eucaryota. Little is known about ncRNA in thermococcal archaea except for the eukaryotic-like C/D and H/ACA modification guide RNAs.

**Results:**

Using a combination of *in silico *and experimental approaches, we identified and characterized novel *P*. *abyssi *ncRNAs transcribed from 12 intergenic regions, ten of which are conserved throughout the Thermococcales. Several of them accumulate in the late-exponential phase of growth. Analysis of the genomic context and sequence conservation amongst related thermococcal species revealed two novel *P*. *abyssi *ncRNA families. The CRISPR family is comprised of crRNAs expressed from two of the four *P*. *abyssi *CRISPR cassettes. The 5'UTR derived family includes four conserved ncRNAs, two of which have features similar to known bacterial riboswitches. Several of the novel ncRNAs have sequence similarities to orphan OrfB transposase elements. Based on RNA secondary structure predictions and experimental results, we show that three of the twelve ncRNAs include Kink-turn RNA motifs, arguing for a biological role of these ncRNAs in the cell. Furthermore, our results show that several of the ncRNAs are subjected to processing events by enzymes that remain to be identified and characterized.

**Conclusions:**

This work proposes a revised annotation of CRISPR loci in *P*. *abyssi *and expands our knowledge of ncRNAs in the Thermococcales, thus providing a starting point for studies needed to elucidate their biological function.

## Background

A plethora of noncoding RNAs (ncRNAs), including small RNAs that bind to proteins or base pair with target RNAs, have been found to operate at all levels of gene regulation ranging from the control of enzymatic activity to the regulation of the initiation of transcription and translation [1-3]. However, whole genome analyses of both prokaryotic and eukaryotic organisms have generally disregarded ncRNA genes. In Bacteria, systematic searches for functional intergenic regions have led to the discovery of more than 200 bacterial trans-acting ncRNAs of about 50 to 500 nucleotides mostly in *E*. *coli *[1,4,5], but also in other pathogenic species [2,6-8]. Functional analysis of these ncRNAs identified many of them as regulators of bacterial stress responses. Furthermore, the recent discovery of riboswitches [9] and RNA-based thermosensors [10] in bacterial 5' untranslated regions (5'UTRs) has enlarged the range of posttranscriptional control of gene expression. In Archaea, computational and experimental analysis lead to the identification and characterization of the homologues of the eukaryotic box C/D- and H/ACA guide snoRNAs, which are involved in modification and maturation of tRNAs and rRNAs in Crenarchaea and Euryarchaea [11-14].

RNomics studies have revealed the occurrence of stable antisense RNAs and the expression of ncRNAs from intergenic regions in *Sulfolobus solfataricus *and *Archeoglobulus fulgidus *[15-17]. *In silico *searches using the *Pyrococcus furiosus *and *Methanocaldococcus janashii *genomes, predicted several novel ncRNAs originating from intergenic regions [18,19]. More recently, transcriptome analysis has revealed several dozen ncRNAs in the halophilic euryarchaeon *Haloferax volcannii *[20,21] and more than two hundred in the methanogenic crenarchaeon *Methanosarcina mazeï *[22]. Among the ncRNAs in *Archeoglobulus fulgidus *[15]*, Sulfolobus solfataricus, Sulfolobus acidocaldarius *[16,23] and *Pyroccocus furiosus *[24], several correspond to ladder-like transcripts issued from repeated genomic sequences or CRISPR loci, [25,26]. The CRISPR/Cas defense system (for review [27]), identified in most archaeal genomes as well as many bacterial genomes, provides acquired immunity against viruses and plasmids by targeting nucleic acid in a sequence-specific manner [28,29]. The anatomy of a CRISPR locus has been defined as an array of short direct repeats of 20 to 50 base pairs, often containing palindromic sequences [30,31]. Irrespective of the precise mechanism of the defensive action of CRISPR/Cas systems, there is a consensus that transcription of the CRISPR cassette initiates in or near the leader sequence followed by processing by the Cas proteins of the RNA precursor into fragments (crRNAs) corresponding to the interval of the repeats [24].

Several stable RNAs in the Archaea, including the box C/D and H/ACA guide RNAs, the ribosomal RNAs and RNase P, form ribonucleoprotein complexes (RNPs) with the multifunctional L7Ae protein that binds to the Kink-turn (K-turn) [17,32,33] or the related Kink-loop (K-loop) RNA structural motif [34]. These widespread motifs [35] provide a platform for assembly of RNPs or, in the case of the S-adenosylmethionine and lysine riboswitches, orient strands that base pair to form pseudoknots [36,37].

The goal of this study was to identify and characterize novel ncRNAs in *Pyrococcus abyssi*, a thermococcal archaeon (hyperthermophilic and anaerobic member of the euryarchaeal phylum), which is one of the first archaea whose genome was sequenced [38]. Stable noncoding RNAs that have been identified in *P*. *abyssi *are ribosomal RNAs (1 16S, 1 23S, and 2 5S genes), RNase P (1 gene), tRNAs (46 genes), 7S RNA (1 gene), H/ACA guide RNAs (7 genes) and C/D guide RNAs (59 genes). Most of these genes have a significantly higher (G+C) content compared to the rest of the genome, which is AT rich. The (G+C) content of the P. *abyssi *genome is 44% compared to 66% and 70% in rRNA and tRNA genes, respectively. Considering the availability of several related thermococcal genomes and the AT rich character of *P*. *abyssi *genome, we performed computational searches for novel ncRNAs. We clustered InterGenic Regions (IGRs) based on primary and secondary structure features, and sequence conservation in other thermococcal genomes. Northern blotting showed that 24 out of the 82 selected IGRs are transcribed. Additional primer extension and Circular Rapid Amplification of cDNA Ends (C-RACE or CR-RT-PCR) experiments and *in silico *analysis showed that twelve of these transcripts have characteristics of regulatory ncRNA: three are from CRISPR cassettes, three are from mRNA 5'UTRs and six are from intergenic regions. Altogether, this study allowed us to define two novel families of ncRNA in *P*. *abyssi*, the CRIPSR and the 5'UTR derived ncRNAs.

## Results

### Detection of novel ncRNAs

The *Pyrococcus abyssi *IGRs were screened using complementary approaches (Additional file 1, Figure S1). The first one, which takes into account specific characteristics of the *P*. *abyssi *genome, recovered 73 GC-rich regions in 67 IGRs including 51 known ncRNA genes and 22 novel regions. The high number of known ncRNA genes found by this approach confirms the interest in using AT-rich genomes in computational search for ncRNA genes. The second comparative approach, which highlights sequence conservation between four closely related *pyrococcal *and *thermococcal *species, produced 106 regions similar to at least one region of another thermococcal genome. The 106 regions cover 95 IGRs of the *P*. *abyssi *genome. Based on alignment features, 65 regions were selected manually from both sets of candidates as described in the Methods section. Fourteen regions encoding novel putative ncRNAs were found by both approaches **(**Additional file 1, Figure S1). To refine our search, 73 regions predicted by both approaches were considered with regard to other criteria as described in the Methods section including free energy of RNA folding, presence of highly structured hairpins, and RNA motifs such as K-turns and K-loops [34,39] as well as similarities between the *P*. *abyssi *genome and all other available archaeal genomes. Altogether, 82 regions were selected as putative genes for ncRNA (Figure S1, Table S1). To test whether all these regions express detectable transcripts, we performed Northern blot analysis with strand-specific direct and reverse oligonucleotide probes (Additional file 2, Table S1), and RNA extracted from *P*. *abyssi *in exponential, entry into stationary or stationary phase cultures. Signals were detected in 24 out of the 82 regions. Five regions generated transcripts up to 700 nt in length (data not shown). They were excluded from further analysis since they were likely to correspond to regions within polycistronic mRNA. We focused on the 19 regions transcribing RNAs shorter than 500 nt. Seven regions corresponded to the H/ACA RNA genes recently annotated in the *P*. *abyssi *genome [13]. The remaining 12 regions were clustered into four categories (Table 1). The first includes two CRISPR loci expressing three 60 nt ncRNAs (crRNAs), the second includes four unique loci in the *P*. *abyssi *genome expressing RNAs ranging in size from 50 to 100 nt, the third includes three repetitive loci conserved throughout thermococcal genomes expressing RNAs ranging in size from 130 to 220 nt, and the fourth includes two repetitive loci specific to *P*. *abyssi *expressing RNAs ranging in size from 145 to 343 nt. More often than not, abundance of the expressed RNAs was growth-regulated with the highest amounts detected in entry into stationary phase. In many cases, several RNA species arose from the same locus suggesting the processing of a primary transcript to secondary transcripts.

**Table 1:** Novel ncRNAs validated in this study

Name	Beginning	End	length(nt)	5'-start	Promoter	Adjacent genes	Strand	Conservation	Prediction
*CRISPR locus*									

Cr 1-1	149430	147916	60	149408	Cons (53 nt)	PAB0095/PABt02	→←←	All^§^	H/R
		
Cr 4-4	1760062	1761854	60	1760285	Cons (53 nt)	PAB1170/PaBt46	←→→		H/R
								
Cr 4-12			60	1760832					

*Conserved unique locus*									

sRk11	197248	197534	50	197484	TATA	PAB2227/PAB2402	←←←	All	H/R

sRk28	527697	527833	70**/**100	527798	-	PAB1992/PAB1991	←←←	All except *Tsi*	H/R

sRk33*	636804	636904	100	636804	Cons	PAB1916/PAB0465	←→→	All	H/R

sRk61	1348633	1348700	60/95	1348615	-	PAB1455/PAB0921	←→→	*Pho*	R

*Conserved repetitive locus*									

sRk48	985849	986002	130	985805	Cons	PAB0686/PAB0686.1n	→→→	*Pho, Pfu, Tko, Tsi*	H/R

sRk49	1067535	1067886	150/220	1067923	TATA (60 nt)	PAB0740/PAB0741	→←→	*Pho*	R

sRk52	1104008	1104286	130/190	1104056	Cons	PABt30/PAB0766	←→→	*Pho, Pfu, Tko, Tsi*	H/R

*Specific repetitive locus*									

sRkB	809887	810256	216/343	810048	Cons	PAB1794/PAB0571	←→→		H

sRkC	1612986	1613347	145/220	1613195	Cons	PAB1080.4n/PAB1080.5n	←←←		H

The positions indicate the beginning and the end of each predicted region. The RNA lengths are based on Northern blot analysis. The 5' ends of ncRNAs were determined by primer extension experiments (Additional file 3, Figure S2). Promotors were predicted based on search of entire (Cons: BRE/TATA) or partial (TATA) motif consensus located 15 to 30 nt (otherwise pointed out in parenthesis) upstream of the 5' transcription start. Conservation among the 7 annotated thermococcal genomes: *P*. *abyssi, P*. *horikoshii (Pho), P*. *furiosus (Pfu), T*. *kodokarensis (Tko), T*. *sibiricus(Tsi), T*. *gammatolerans *and *T*. *onnurineus*. Prediction tools are denoted for each region: bias composition (H) and comparative analysis (R). ^§ ^CRISPR 1 and 4 direct repeats are conserved. * sRk33 is annotated as SscA in *P*. *furiosus *[22].

### Annotation and expression of *P*. *abyssi *CRISPR loci

**Figure 1:**
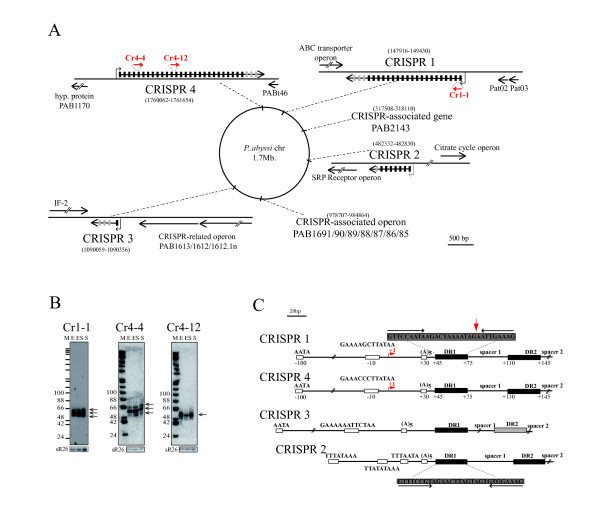
**Expression of *P*. *abyssi *CRISPR cassettes**. (A) Genomic locations of *P*. *abyssi *CRISPR loci. CRISPR cassettes and CRISPR-related operons encoding *cas *and *cmr *genes mapped on the circular *P*. *abyssi *chromosome. Orientations of CRISPR cassettes and adjacent genes are denoted. Depicted gene coordinates are based on the available completed genome of *P*. *abyssi *GE5. Direct repeats and degenerate direct repeats are indicated by black and grey boxes, respectively. Promoters upstream of CRISPR cassettes are denoted by arrows. Cr1-1, Cr4-1 and Cr4-12-RNAs are indicated in red. (B) Stable transcripts detected by Northern blot from CRISPR 1 and CRISPR 4 in exponential growth phase (E), entry into stationary phase (ES) or stationary phase (S). Arrows on the right indicate discrete RNA transcripts. The 5' end-labeled digest of Φx174 DNA with HinfI served as length nucleotide marker (M). The box C/D guide RNA, sR26, served as a loading control (bottom panel). (C) Detailed features of the CRISPR leader sequences drawn to scale with the direct repeat sequence of the two CRISPR families. Positions are relative to the transcription start (+1) of the CRIPSR precursor (preCr) indicated by a red arrow. The palindromic sequences in the direct repeat (DR) are represented by two inverted arrows. The 5' end of CR1-1 is indicated by a red arrow in the DR1 sequence. The -10 and +30 boxes indicate BRE/TATA promoter sequences and a conserved 5 nt poly A sequence, respectively.

The comparative computational screen selected the four CRISPR loci annotated recently in the *P*. *abyssi *genome [40]. A careful sequence inspection of these cassettes revealed an erratic number of repeats and spacers in the *P*. *abyssi *CRISPR loci. Based on recent observations showing that new spacers are integrated mainly on the side proximal to the leader sequence of the CRISPR cassette and that degenerated direct repeats accumulate at the distal 3' end of the cassette [29], we revised the annotation of the *P*. *abyssi *CRISPRs (Figure 1A). The CRISPR 1 (encoded on the reverse strand) and CRISPR 4 (encoded on the sense strand) are composed of 23 and 29 direct repeats and spacers, respectively, with similar 30 nt direct repeat sequences. The observation that the penultimate and ultimate 3' end-direct repeats are degenerate by one to six mismatches confirms the orientation proposed by the UCSC archaeal genome browser [41], which differs from the orientation proposed by [40]. The atypically short CRISPR 3 locus contains only four spacers with degenerate direct repeat sequences related to CRISPR 1 and 4. The CRISPR 2 cassette encompassing eight spacers is distinguished by the sequence of its 29 nt direct repeats. In order to position the degenerate direct repeat (four mismatches) at the end of the CRISPR cassette, as in the majority of the CRISPR loci [27], we propose that CRISPR 2 is encoded by the reverse strand as opposed to previously reported annotations (UCSC archaeal genome browser and [40]).

No signals were detected by Northern blotting with strand-specific direct or reverse probes against sequence matching spacers 2 and 7 of CRISPR 2 and spacer 2 of CRISPR 3 suggesting that these loci are not transcribed (data not shown). This tentative conclusion was confirmed by more sensitive tests involving primer extension analysis to map 5' ends of the CRISPR precursors and to identify promoters (see below). In contrast, probes against sequences matching spacer 1 of CRISPR 1 and spacers 4 and 12 of CRISPR 4 allowed the detection of transcripts in all three growth phases (Figure 1B; Table 1). The approximately 60 nt length of CRISPR-derived RNAs, named hereafter Cr1-1, Cr4-1 and Cr4-12, are of the size reported for crRNAs corresponding to a spacer sequence and part of the direct repeat [16,24]. Moreover, primer extensions with total RNA extracted from cells in entry-stationary phase (Additional file 3, Figure S2A) accurately identified their 5' ends (Figure 1C), which correspond to the dicing site reported for the *P*. *furiosus *endoribonuclease Cas 6 [42]. Since only single reverse transcription arrests are observed (Additional file 3, Figure S2A) even though multiple Northern blot signals are detected (Figure 1B), we propose that length heterogeneity results from partial or incomplete 3'end processing. In conclusion, the CRISPR 1 and 4 cassettes are transcribed to produce small crRNAs in *P*. *abyssi*. No signals corresponding to precursor or other intermediates were detected by Northern hybridization (Figure 1B). Nevertheless, primer extensions with oligonucleotides hybridizing to the 5' junction of their respective first direct repeat resulted in the detection of an RNA precursor and thus permitted the identification of the transcription starts of the CRISPR 1 and 4 RNA precursors (preCr1 and 4, respectively) (Figure 1C & Additional file 3Figure S2A). Similar experiments with specific primers of CRISPR 2 and 3 did not reveal any RNA precursor confirming that CRISPR 2 and 3 are not expressed. Signals were not detected in Northern hybridization with probes against the ultimate spacer 22 of CRISPR 1 and spacer 28 of CRISPR 4, which harbor degenerate repeats. These results suggest that the direct repeats are important for the processing and/or stability of the mature crRNAs. To identify transcription signals, we analyzed the 200 bp region upstream of the first direct repeat of each *P*. *abyssi *CRISPR cassette. This analysis recognized conserved AT rich motifs arguing for the presence of promoters. The CRISPR 2 leader sequence has three AT-rich and one A-rich element upstream of the first direct repeat, not corresponding to any typical *P*. *abyssi *consensus promoter sequence as defined in [38]. In contrast, the CRISPR 1 and 4 cassettes have consensus promoter sequences 10 nt upstream of the experimentally determined 5' starts of preCr1 and 4 (Figure 1C). Two additional short elements at position +30 and -100 relative to the transcription starts were detected in CRISPR 1 and 4. These sequences are also observed in other thermococcal CRISPR cassettes at the same distance from the first direct repeat (data not shown). It should be noted that the general organization of the CRISPR 3 leader is similar to those of CRISPR 1 and 4 except for the distance between the +25 A-stretch and the first direct repeat (Figure 1C). This feature might account for the absence of transcription of the CRISPR 3 locus.

### Expression of unique intergenic regions conserved among thermococcal genomes

**Figure 2:**
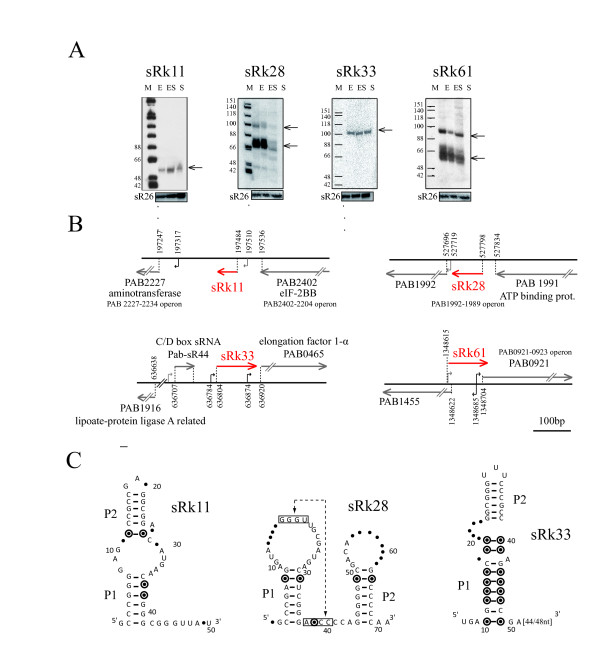
**Four ncRNAs from unique intergenic region conserved among thermococcal genomes**. (A) Transcripts detected by Northern blot from regions with a unique locus in the *P*. *abyssi *genome. Source of RNAs, markers and sR26 loading control are as indicated in Figure 1B (B) Gene maps drawn to scale. Transcripts and predicted genes are symbolized by red and grey arrows, respectively. Consensus promoters and TATA-box sequences (as defined in Materials and Methods) are indicated in black and grey, respectively. The 5' start of each novel RNA was experimentally determined (Figure S2). (C) Secondary structure models with conserved nucleotides indicated by A, C, G and U, variable nucleotides conserving pairing by circled dots and variable nucleotides by dots. A putative pseudoknot in sRk28 is denoted by a dotted line. The underlined sequence in sRk33 indicates the putative promoter of PAB0465.

Four ncRNAs were clustered in this category (Table 1). For three of them, sRk11, sRk33 and sRk61, expression is detected at about the same intensity in all growth conditions whereas sRk28 is clearly growth phase-regulated with little or no detectable RNA in stationary phase (Figure 2A). Remarkably, the genomic contexts of sRk11, sRk28 and sRk33 are similar, all being flanked by ORFs transcribed in the same direction. Both, sRk33 and sRk61 cover predicted promoter sequences of the downstream annotated genes (Figure 2B). The respective 5' ends (Table 1) determined by primer extension experiments (Additional file 3, Figure S2B) are at an acceptable distance from the predicted promoter elements (BRE/TATA and TATA boxes) for sRk11 and sRk33, and overlap a predicted TATA box for sRk61 (Figure 2B). No such sequence is found upstream of sRk28 which might suggest that its transcription depends on the upstream gene PAB1991 since this region is predicted to form an operon that includes PAB1992. Except for sRk61, these ncRNAs are extremely well conserved in sequence and structure throughout other thermococcal genomes (Additional file 4, Figure S3A). The proposed RNA secondary structures, based on RNAfold and multiple alignment covariation analysis, highlight compensatory variations that maintain hairpin structures and, for sRk28, a potential pseudoknot structure (Figure 2C). Only sRk33 was predicted and validated in a previous comparative analysis in *P*. *furiosus *(referred to as SscA in [19]). sRk33 has a predicted secondary structure with a basal P1-stem with a high proportion of co-variations, an apical P2 stem conserved in sequence, and a 3' end sequence composed of A and U rich stretches that do not fold into a stable secondary structure (Figure 2C). Altogether, these characteristics suggest that sRk11, sRk28 and sRk33 might have conserved cellular functions in the Thermococcales.

### Expression of repetitive intergenic regions conserved among thermococcal genomes

Among the three ncRNAs clustered in this category (Table 1), sRk49 shows sequence similarities with two additional regions in *P*. *abyssi *(49.2 and 49.3) and three in *P*. *horikoshii* (Additional file 5, Figure S4A). Sequence similarities preclude the design of specific probes to distinguish between sRk49 and 49.2. Northern blot signals provided evidence for a growth stage-specific transcription pattern with the most abundant species migrating about 150 nt. These transcripts could arise from either of the loci harboring an upstream TATA-like promoter (sRk49 and 49.2) (Additional file 5, Figure S4B & C). CR-RT-PCR experiments failed to detect primary transcripts, but suggested that antisense transcripts are expressed from the sRk49 operon (data not shown). Specific RNA structures or RNA modifications could have impeded the accurate amplification of the primary transcripts. Moreover, related sequences in the *P*. *horikoshii *genome are all clustered with CRISPR cassettes showing an organization comparable to that observed for the 49.2 region. All sRk49 variants are atypical in that they contain multiple short polyA stretches making RNA folding into stable helical structures implausible.

**Figure 3:**
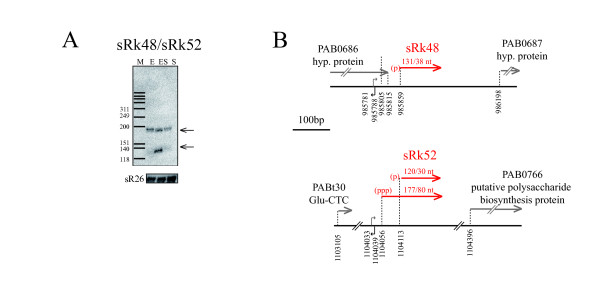
**sRk48 and sRk52 from repetitive intergenic regions conserved among thermococcal genomes**. (A) Detection of sRk48/sRk52 by Northern blot. Source of RNAs, markers and sR26 loading control are as indicated in Figure 1B. (B) Gene maps drawn to scale. Transcripts are specified in red along with the nature of their respective 5'end, (ppp) or (p) for tri- or mono-phosphate, respectively.

**Figure 4:**
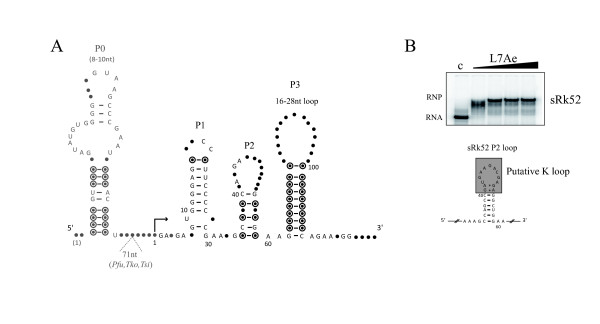
**Secondary structural model of sRk48/52 RNA candidates**. (A) The model is based on sequence alignments performed with the genomic sequences in *P*. *abyssi *and extended to the four thermococcal genomes (*P*. *horikoshii, P*. *furiosus, T*. *sibiricus and T*. *kodokarensis*) (Additional file 6 Figure S5). (B) EMSA (upper panel) of the 5' end labeled 130 nt sRk52 RNAs in presence of L7Ae protein at 50, 100, 200 and 400 nM. The first lane (c) corresponds to the RNA without protein. The putative K-loop structure in the P2 stem is indicated (bottom panel).

The two other ncRNAs of this category are sRk48 and sRk52 (Table 1, Figure 3). They were identified independently in our initial computational search and are part of a set of six similar genomic sequences in *P*. *abyssi*. The similarities extend to all thermococcal genomes with four, three, two and one copies in *P*. *furiosus, P*. *horikoshii, T*. *sibiricus and T*. *kodokaraensis*, respectively (Additional file 6, Figure S5). The sequence similarities precluded the design of specific probes for each locus. Therefore, the signals detected in the Northern blot in Figure 3A could arise from any of the six repeated genomic regions. In order to identify the transcribed regions, a CR-RT-PCR experiment was performed. Most clones corresponded to the 130 nt transcript with a majority matching sRk52 and a minority sRk48 (Figure 3B). They shared similar 5' ends and variable 3' ends. In addition, a small percentage of clones corresponded to longer variants of sRk52 (177/180 nt) mapping a transcription initiation site 22 nt downstream a predicted BRE/TATA box promoter (Additional file 5, Figure S4). The shorter 5'-monophosphorylated sRk52 transcript is likely to have arisen from maturation of the longer primary transcript. Interestingly, RNAfold and multiple sequence alignments suggest a highly conserved RNA secondary structure within the Thermococcales (Figure 4A). Many nucleotides, particularly in the P0 and P3 stems, support co-variations arguing for structural conservation. The majority of the cloned CR-RT-PCR fragments contain sequences corresponding to the P1, P2 and P3 stem-loops whereas a minority includes the P0 stem-loop in their 5' part (Figure 4A). The P1 stem-loop is unusual since its primary sequence is highly conserved throughout the Thermococcales giving this structure not only a high propensity to form, but also suggesting an important cellular function. Inspection of the P2-loop of sRk52 suggested that it forms a K-loop, which is consistent with the gelretardation of 5'end-labeled sRk52-transcripts in presence of the recombinant *P*. *abyssi *L7Ae protein (Figure 4B). The P3-loop is variable and can be extended into a stem structure for the two *P*. *abyssi *sRk48-like sequences.

### Expression of repetitive intergenic regions specific to *P*. *abyssi*

**Figure 5:**
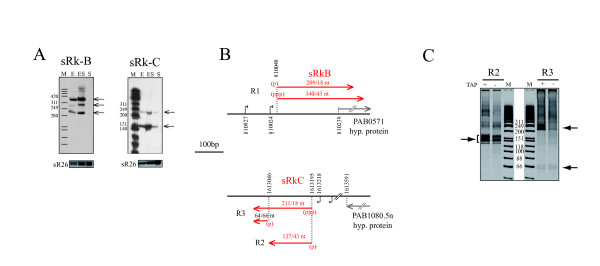
**sRkB and sRkC RNA candidates from repetitive intergenic regions specific to *P*. *abyssi *genome**. (A) Detection of sRkB and sRkC by Northern blotting. Source of RNAs, markers and sR26 loading control are as indicated in Figure 1B. (B) Gene maps drawn to scale. (ppp) and (p) represent tri- and mono-phosphate 5' ends, respectively. Three CR-RT-PCRs were performed using primer pairs designed to detect sRkB (R1, top panel) and sRkC (R2 and R3, bottom panel). In R2 and R3, two different primer pairs used to permit the complete mapping of the sRkC transcripts. (C) Native 6% PAGE of the CR-RT-PCR products using specific primers (Additional file 7 Table S2). Above the lanes, -/+ indicates mock control or treatment with tobacco acid pyrophosphatase (TAP).

Amounts of the two ncRNAs of this category, sRkB and sRkC, were highest upon entry into stationary phase (Figure 5A; Table 1). Because these loci (Figure 5B) have significant sequence similarities to four other regions dispersed in the *P*. *abyssi *genome, we performed additional experiments to determine if the other loci were transcribed. Despite the sequence similarities, we could design oligonucleotide probes specific for each repetitive locus and perform additional Northern blot probing (data not shown), CR-RT-PCR experiments (Figure 5B and 5C) and primer extension analyses (Additional file 3, Figure S2B). From these data, the sRkB and sRkC loci were shown to be the only regions transcribed. In order to identify the 5' and 3' ends of these transcripts, three distinct CR-RT-PCR experiments were carried out. One pair of primers specific of the sRkB locus (R1) and two pairs of primers specific of the sRkC locus (R2 and R3) were chosen to characterize the overall RNAs expressed at these loci. The majority of the R1 clones correspond to transcripts from the sRkB locus harboring an identical 5'end and a variable 3'end of which half ended between position 209 to 218 nt and half between position 340 to 343 nt (Figure 5B and Additional file 4, Figure S3B). The length of sRkB RNAs as determined by CR-RT-PCR, corresponds to the sizes of the transcripts detected in Figure 5A. Moreover, sequence analysis revealed two typical promoter consensus sequences 24 nt and 179 nt upstream of the experimentally determined 5' end (Figure 5B and Additional file 4Figure S3B). Interestingly the 3' extremities of all the RNA species are in the first 25 to 150 nucleotides of the PAB0571 ORF. Additional primer extension analysis with a specific probe within PAB0571 suggests that a unique RNA precursor is transcribed from this locus and subsequent processing events including 3' end trimming generate the different sRkB RNA species (data not shown). The R2 and R3 CR-RT-PCR analyses permitted the identification of three stable RNA species from the sRkC locus (Figure 5B). The two longer RNA species of roughly 137 nt (majority of the sequenced R2 clones) and 211 nt (half of sequenced R3 clones) share exactly the same 5' extremity. The 5' ends of the shorter 64/66 nt RNA species are positioned 149 nt downstream (minority of sequenced R3 clones). In addition, primary transcripts were distinguished from processed ones by omission of treatment of the RNA preparations with tobacco acid pyrophosphatase (TAP) (Figure 5C). The 5' ends of 211/18 nt-RNA species are mostly triphosphorylated. As in the case of sRkB, a promoter sequence is found at an acceptable distance (24 nt) from the 5' end (Figure 5B and Additional file 4Figure S3B). RNA species transcribed from the sRkC locus could result from 5' and 3' end processing events of the primary 211/218 nt long transcripts resulting in precise 5' monophosphorylated ends and variable 3' ends. However, it should be noted that the 64/66nt RNA species were not detected in Northern blot analysis with specific probes (data not shown) suggesting that they are in low abundance in our RNA preparations. In contrast to the sRkB locus, the sRkC locus is located in a long intergenic region. The 3' end of PAB1080.5n, the nearest ORF, is located approximately 400 bp upstream of the 5' end of sRkC (Figure 5B).

As mentioned earlier, the sRkB and sRkC loci are part of a set of six similar sequences that had no counterparts in other thermococcal genomes and are therefore specific to *P*. *abyssi*. Consistent with their lack of expression, the BRE/TATA promoter sequences are missing in the other four other *P*. *abyssi *loci (Additional file 4, Figure S3B). Strikingly, one of these loci corresponds to the 3' end of the PAB1452 open reading frame. This ORF has been identified as a 'single OrfB element' of the IS*605*/IS*200 *family of transposases [43].

**Figure 6:**
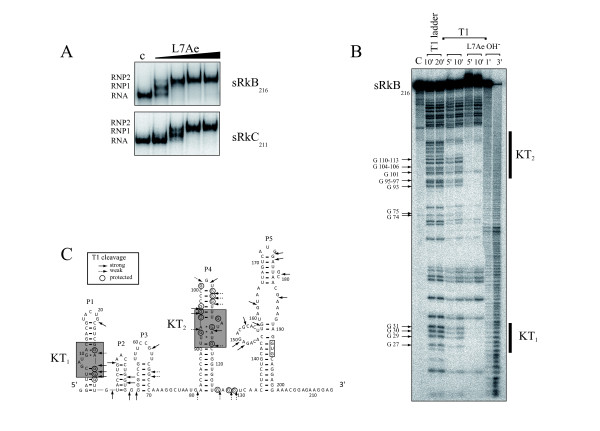
**sRkB and sRkC RNAs bind to multifunctional ribosomal L7Ae protein**. (A) EMSA of the 5'end-labeled 216 nt sRkB (sRkB_216_) and 211 nt sRkC (sRkC_211_) RNAs in presence of L7Ae in the same condition as in Figure 4B. (B) Footprinting of L7Ae on 5' end labeled sRkB_216_. No reaction control (C), digestion under denaturing conditions with RNAse T1 (T1 ladder) or alkaline hydrolysis (OH^- ^ladder) are as indicated. The four middle lanes reveal cleavages by RNase T1 in presence or absence of the L7Ae protein (400 nM). Protected G residues are indicated. (C) Experimentally derived secondary structure of sRkB with RNase T1 cleavages in absence of L7Ae (strong cleavages with black arrows and weak with dotted arrows) and protected guanosines in presence of L7Ae (circles). The two kink turn structures (KT_1 _) and (KT_2_) are boxed in grey.

Interestingly, in our initial computational characterization, sRkB and sRkC were predicted to fold into a structure with a K-turn RNA motif. To test if sRkB and sRkC form a K-turn, we performed gel retardation assays with recombinant *P*. *abyssi *L7Ae (Figure 6A). This assay reveals at least two distinct ribonucleoprotein complexes (RNPs) suggesting that more than one L7Ae protein binds to the sRkB and sRkC RNAs. To elucidate these RNA-protein interactions, footprint experiments using RNase T1 protection were performed with radioactively labeled 216 nt sRkB transcript (Figure 6B). Two sets of clustered guanosines are protected from RNase T1 digestion in the presence of L7Ae, suggesting K-turns in stems P1 (KT_1_) and P4 (KT_2_) (Figure 6B). Further support for the existence of KT_1 _and KT_2 _was obtained by mutations of the sheared GA base pairs, which are the hallmark of the K-turn. In this case, gel retardation assays showed that the shift with recombinant *P*. *abyssi *L7Ae was abolished (data not shown). Sequence alignment (Additional file 4, Figure S3B) and RNA-fold predictions support the RNA secondary structure shown in Figure 6C. Altogether, our data reveal that several transcripts expressed from the sRkB and sRkC loci have K-turn motifs that might be important for their cellular function. Another well-known RNA structural motif, a loop E [39], might form in the upper part of P5. However, since there is no specific ligand that interacts with loop E, validation of this fold requires a detailed structural analysis. It is interesting to note that the initiation codon for the PAB0571 is part of the P5 stem of sRkB suggesting a functional link with the expression of the downstream PAB0571.

## Discussion

### Discovery of novel ncRNAs by combined bioinformatic approaches

The rationale for carrying out a search of ncRNAs in *P*. *abyssi*, the best studied Thermococcale, was that with the exception of the box H/ACA [13] and the box C/D [12] noncoding guide RNA families, few thermococcal ncRNA families have been described. Our study provides evidence for the growth-regulated expression of 12 novel ncRNAs in the *P*. *abyssi *genome consisting of three crRNAs from CRISPR loci, four ncRNAs from unique loci conserved throughout Thermococcales of which one appears to be a homologue of the *P*. *furiosus *SccA RNA [19], three ncRNAs from repetitive loci conserved throughout Thermococcales, and two ncRNAs from repetitive loci specific to *P*. *abyssi*. Since small proteins (sproteins) of less than 50 amino acids have been largely disregarded in genome annotations [44], we cannot exclude the possibility that the ncRNAs identified here encode sproteins or peptides. We therefore searched for open reading frames within the ncRNA sequences starting with AUG, GUG or UUG, and ending with UGA, UAG or UAA. For several of the ncRNAs, it was possible to find small ORFs of 25 to 40 amino acids. The only significant ORF identified corresponds to the distal portion of a putative transposase CDS (see below). We also analyzed the GC content of the ncRNAs. We found that it ranges from 33% to 66%. This large spectrum is consistent with the GC content for known ncRNAs (low for C/D guide RNAs, high for tRNAs and rRNAs). Since the computational analyses were restricted to intergenic regions, no *cis*-encoded antisense ncRNA complementary to ORFs, as identified in transcriptome analyses of *Sulfolobus solfataricus *and *Methanosarcina mazei *[22,45] could be predicted. Below, experimental and genomic features of the new *P*. *abyssi *ncRNA families are discussed.

### Processing of ncRNAs

In this study, transcripts of different length were identified at one locus suggesting that RNA processing occurs in *P*. *abyssi*. The sRkB, sRkC, sRk48 and sRk52 primary transcripts with triphoshorylated 5' ends are processed into shorter 5' end monophosphorylated transcripts through maturation reactions (Figure 3C and Figure 5C). For example, the 3' end heterogeneities observed for sRkB, sRkC, sRk48 and sRk52 RNAs could arise from trimming of the primary transcript by the 3' to 5' exonucleolytic activity carried by the *Pyrococcus *exosome [46]. The 5' monophosphorylated RNAs could result from endonucleolytic or pyrophosphohydrolytic activity. Previous examples of processing in *Pyrococcus *were limited to tRNA processing by RNase P [47] and crRNA maturation by the Cas 6 endonuclease [42,48]. Recent results suggest that the 5' to 3' exonucleolytic activity of RNase J homologues may also be involved in RNA processing in Euryarchaea and Crenarchaea [49,50].

### CRISPR-derived RNAs

Our detection of crRNAs is the first demonstration of the existence of an active CRISPR defense system in *P*. *abyssi*. The CRISPR loci 1 and 4 appear to be expressed independently of growth phase in our experimental conditions. In agreement with the findings in *P*. *furiosus *[51], no antisense transcription of these CRISPR loci was detected (data not shown) excluding the possibility that double strand RNA intermediates are formed. This contrasts with the report that RNA is transcribed from the complementary spacer strand in *S*. *acidocaldaricus *[52]. This difference could reflect a specific feature of the Sulfolobales or a technical issue regarding the sensitivity of the Northern blots analysis used in the studies of *P*. *furiosus *and *P*. *abyssi*. Based on our data, we propose a new annotation for the four *P*. *abyssi *CRISPR arrays that differs from earlier proposals (UCSC archaeal genome browser and [40]). The presence of a typical consensual promoter in the leader sequences of CRISPR 1 and CRISPR 4 correlates with the detection of CRISPR-derived RNAs from these loci. It has been reported that CRISPR cassettes expressing crRNAs provide acquired immunity [28,53,54]. It should be noted that short regions of spacers 7 and 19 of CRISPR 1 (14 bp and 17 bp, respectively) are complementary to two coding regions of PAV1, a virus that infects *P*. *abyssi *(data not shown) [55]. No other similarities were observed between CRISPR 1 and 4 spacer sequences and known *P*. *abyssi *mobile genetic elements [56,57]. The silent CRISPR 2 and 3 loci differ from the expressed cassettes in several ways. First, the CRISPR 2 locus has divergent direct repeat and leader sequences. The CRIPSR 3 locus is atypical by its leader sequence, the reduced number of repeats, and the sequence degeneracy of its repeats, suggesting that it is a relic of an active CRISPR cassette as mentioned in [27,28,58]. In general, CRISPR cassettes are physically linked to a cohort of conserved *cas *or *cmr *genes, in varying orientation and order, that encode CRISPR-associated proteins (reviewed in [24,27,31]). It is interesting to note that the only *cas *gene (Cas6, PAB1613) in the vicinity of *P*. *abyssi *CRISPR cassettes is located upstream of CRISPR 3 (Figure 1A). In the *P*. *abyssi *genome, all the other genes encoding Cas/Cmr core proteins are grouped into an operon located far from any CRISPR locus (Figure 1A). Further studies will be required to elucidate the mode of action and the dynamics of the CRISPR/Cas system in *P*. *abyssi*.

### 5' UTR-derived ncRNAs

Distinct RNA species are not always indicative of independently synthesized RNAs. As revealed by RACE experiments and analysis of genomic context, several ncRNAs (Table 1) seem to derive from mRNA leaders. This class of ncRNAs appears to originate from maturation of longer transcripts, as we demonstrated for sRkB and propose for sRk28, sRk33 and sRk61. Stable RNAs derived from RNA leaders were initially identified in *E*. *coli *and some appear to correspond to riboswitch elements such as RFN and THI [59]. It is now well established that 5'UTRs can encompass transcription-termination signals involving riboswitch structures [60,61]. In Archaea, it has recently been suggested that riboswitches may also exist. Based on comparative genomics, *crcB *has been found in Archaea and Bacteria, and *TPP *and *flp *in Euryarchaea [60,62]. The *crcB *element was found within our set of 82 candidates (Additional file 2, **Table S1**), but ncRNA corresponding to this element was not detected by Northern blotting under the conditions employed in this study. To date, no biological evidence showing gene regulation by riboswitches has been reported in Archaea. Nevertheless, the conserved 70 nt sRk28 shares several feature with the bacterial preQ1 riboswitch [63,64]. PAB 1992, adjacent to sRk28, is predicted to encode an ATPase with a queosine synthesis-like protein domain (QueC). The growth-regulated sRkB RNA is transcribed from a locus adjacent to PAB0571, which encodes a putative ATPase. In bacteria, the expression of several ATPase-like proteins is controlled by *cis*-mechanisms involving the SAM-I riboswitch. It is interesting to note in *Listeria monocytogenes *that this type of riboswitch also acts *in trans *as a small ncRNA to regulate expression of the virulence factor PrfA [7]. Moreover, our data strongly suggest that sRkB has the propensity to form K-turn structures, which are found to be functional RNA motifs involved in the assembly of ribonucleoprotein complexes and in the orientation of pseudoknot interactions in the aptamer structures of the SAM-I and lysine riboswitches [36,37]. Therefore, sRkB could have a specific function in the *P*. *abyssi *cell that involves the regulation of the expression of the putative ATPase encoded by PAB0571. We speculate that sRk28 and sRkB may be representative of a novel family of archaeal ncRNAs produced by transcription attenuation or RNA processing. Finally, the promoter-associated sRk33 and sRk61 could be related to the recently discovered ncRNAs associated with transcription starts sites reported in higher eukaryotes [65,66], in yeast [67,68] and in *Salmonella enterica *serovar typhi [69]. This class of ncRNAs is thought to interfere with transcription of the downstream promoter by *cis*-mediated occlusion or by a *trans*-mechanism.

### Transposase-related ncRNAs

In this study, sRk48, sRk52, sRkB and sRkC ncRNAs, which are transcribed from repetitive loci, were shown to be related to a CDS annotated as an orphan OrfB element of the IS*607 *and IS*200*/*605 *family, [43]. For example, in the case of sRk48 and sRk52, similar sequences correspond to 3' end coding regions annotated as partial OrfB-like IS*607 *transposase in *P*. *furiosus *and *T*. *sibiricus*. In bacteria, it has been shown that IS*10 *transposition events are controlled by a complementary *cis*-encoded antisense ncRNA [70], but this seems unrelated to our observations since the ncRNA sequences matched the sense sequence of the 3' end of the annotated ORFs. However, it is pertinent to note that a link between ncRNAs and transposase ORFs was already observed in *Sulfolobus solfataricus *[16,17] and in Salmonella [71]. Moreover, in *Sulfolobus solfataricus *[17] it was shown that several small RNAs linked to annotated transposase ORFs could bind the multifunctional ribosomal protein L7Ae through recognition of an RNA kink turn as is the case for sRkB, sRkC and sRk52.

## Conclusions

Two novel *P*. *abyssi *ncRNA families, the CRIPSR and the 5' UTR-related ncRNAs are described in this study. We certainly missed other *P*. *abyssi *ncRNA families because the biased composition screen only identifies GC rich ncRNAs. This is the case for the box C/D guide RNAs with low GC content. Nevertheless, the high number of known ncRNAs including the box H/ACA RNAs found by this approach confirms its relevance in searching for ncRNAs in AT-rich genomes. A limitation to this search, which is a bias due to misannotated ORFs and therefore intergenic regions, could not be excluded since sequence signals for archaeal transcription and translation are not as well defined as their counterparts in Bacteria. The discovery of novel ncRNAs by our combined computational approaches emphasizes the potential diversity of ncRNAs in Archaea, which could be enlarged by global RNomic approaches such as RNAseq technology. This would provide a deeper insight into the *P*. *abyssi *ncRNA world and help improve our knowledge of the specific roles of *P*. *abyssi *ncRNAs and their relationship to the 5'UTRs described in this study.

## Methods

### Computational search

Genome sequences and related annotation files (gbk, .fna and .ptt extensions) were downloaded from the NCBI ftp database for *P*. *abyssi*, *P*. *furiosus*, *P*. *horikoshii *and *T*. *kodakaraensis *genomes. For these four genomes, a comparative analysis of all intergenic sequences was realized using RNAsim as described in [2]. RNAsim searches for conserved sequences and structured regions between different genomes by using Wu-blast 2.0 to select pairwise alignments including conserved sequences (here with W = 7, E < 0.0001) and QRNA [72] to identify in pairwise alignments base substitution patterns that could correspond to a conserved RNA secondary structure. In a final step, RNAsim combines this information to predict loci that are conserved in multiple genomes. In this analysis, all the regions encoding annotated ncRNAs except for the box H/ACA RNA genes were excluded from the set of intergenic regions (46 tRNAs, 1 rRNA operon, 2 5S rRNAs, 59 C/D guide RNAs, 1 RNase P RNA and 1 7S/SRP RNA). GC-rich regions were predicted in *P*. *abyssi *by using the same segmentation approach as previously used by Klein and colleagues to predict new ncRNAs in *P*. *furiosus *[19]. However, our approach differed in that the transition probabilities were adjusted to enrich the number of candidate regions. Additional sequence comparisons were performed on putative ncRNA candidates with Blastn (default values and W = 7) to add to this initial set other *P*. *abyssi *and archaeal homolog sequences. BRE/TATA, consensus boxes 5'_RNNANNTTTAWA_3' and 5'_RAAANNTWWWWA_3', and TATA consensus box 5'_TTWWWWA_3', K-turn and K-loop consensus motifs, inferred from the literature [34,38,39,73], were identified using Patscan [74]. Regions of favorable free energy were computed by setting the free energy threshold such that all tRNAs were found in sliding windows of 150 bp (here threshold=-32.3). Only regions of free energy below the threshold and longer than 50 bp were displayed in ApolloRNA, an extension of the annotation environment Apollo [75], developed to support ncRNA analysis. Highly structured hairpins with minimal hairpin stems of 6 bp (including G-U and U-G pairs) were searched with Patscan. RNA secondary structures were proposed on the basis of multiple alignments of similar regions within the Pyrococcales using Multalin [76] that were improved manually by combining RNAfold predictions and covariations. All data including predictions, motifs and annotations were integrated and visualized in ApolloRNA.

### Oligonucleotides used in this work

Additional file 7 Table S2 list the primers used for Northern blot detection, CR-RT-PCR, primer extension, and the preparation of transcription templates by PCR.

### Preparation of total cellular RNA

*P*. *abyssi *strain GE5 cells were grown as described in [77] at 95°C in Vent Sulfothermophiles Medium. *P*. *abyssi *cells were stopped at three different growth phases: exponential, end of exponential and stationary phases. The exponential and stationary phase cell paste was provided by A. Hecker (Institut de Génétique et de Microbiologie, Paris Sud-Orsay). Entry into stationary phase cell paste was purchased (Reims University, France). Total RNA was prepared from *P*. *abyssi *cell paste by Trizol extraction followed by treatments with DNase RQ1 (RNA-free, Promega), proteinase K and phenol/chloroform extraction followed by ethanol precipitation.

### Northern blotting analysis

Total RNA (10 μg)extracted from cells in exponential growth phase (E), entry into stationary phase (ES) or stationary phase (S), and a 5' [^32^P]-end-labeled denatured PhiX174/HinfI DNA ladder were separated on a denaturing 6% polyacrylamide gel (8M urea, 1 × TBE buffer). Gels were transferred onto Hybond N+ nylon membrane using a Transphor Power Lid (Hoefer) apparatus in 0.5 × TBE buffer. The RNAs were UV cross-linked to the membrane (1200 J/cm²). Prehybridization was carried out for 30 minutes at 50°C in hybridization buffer (5 × SSC, 1 × Denhartd's solution, 1% SDS, 0.05 mg/mL sperm DNA herring). DNA oligonucleotides were designed using Primer designer or Vector NTI and 5' end labeled with [γ-32P] ATP and polynucleotide kinase. Hybridizations were carried out at 50°C for 16 h followed by two washes in 0.1 × SSC, 0.1% SDS buffer at room temperature for 10 min. The blots were analyzed by phosphorimaging (Molecular Dynamics) or autoradiography using MR or MS film (Kodak).

### Primer extension and Circular RACE (CR-RT-PCR)

Primer extension analysis was performed using 10 μg of total RNA prepared from *P*. *abyssi *cells in entry into stationary phase. Total RNA was reverse transcribed at 42°C by AMV reverse transcriptase (Promega) using a 5' end labeled specific primer. CR-RT-PCR was performed with 20 μg of total RNA prepared from *P*. *abyssi *cells in entry into stationary phase, treated with (+) or without (-) 25U of Tobacco Acid Pyrophosphatase (TAP) according to manufacturer's protocol (Epicentre Biotechnologies). RNAs were extracted with phenol/chloroform then precipitated with ethanol. RNA (1 μg) +/- TAP was circularized with 40U of T4 RNA ligase (New England Biolabs), extracted with phenol/chloroform, ethanol precipitated and reverse transcribed by AMV reverse transcriptase using specific primers. After ethanol precipitation, the reverse transcripts were PCR amplified using Crimson Taq (New England Biolabs) and appropriate primers. The products were separated on a 6% native polyacrylamide gel (1% glycerol, 0.5 × TBE), treated with TAP, cloned in pCR^®^II-TOPO^® ^with TOPO-TA Cloning^® ^Kit according to the manufacturer's instructions (Invitrogen) and sequenced by Eurofins MWG Operon. About 100 sequences were analyzed for each CR-RT-PCR experiment.

### *In vitro *transcription

A portion of the intergenic region corresponding to sRk52, sRkB and sRkC, respectively, was amplified by PCR from *P*. *abyssi *genomic DNA using specific primers (Additional file 2, **Table S1**). PCR products served as templates for *in vitro *transcription using T7 RNA polymerase as previously described [34].

### Electrophoretic mobility shift assay (EMSA) and enzymatic structural probing

EMSA was performed using *E.coli *tRNA as a non-specific competitor as previously described [34]. RNA and RNP complexes were separated on a native 6% (19:1) polyacrylamide gel containing 0.5 × TBE and 5% glycerol. Electrophoresis was performed at room temperature at 250 V in 0.5 × TBE running buffer containing 5% glycerol. The gels were dried and visualized using a Fuji-Bas 1000 phosphorImager.

## Authors' contributions

KP performed experiments, participated in their analysis, and participated in the computational screens. AM participated in the computational screen and bioinformatic analysis. NB initiated the computational screen and experimental validation. DR performed experiments and participated in their analysis. CG implemented the computational screens. CG and BCO conceived the project, participated in its design and coordination. AJC, CG, and BCO wrote the manuscript. All authors read and approved the final manuscript.

## Supplementary Material

Additional file 1 **Figure S1**: Strategy for ncRNA candidate predictions. An initial set of candidates resulted from two complementary prediction methods. The bias composition selected 73 regions within 67 IGRs. The predicted regions expressing known ncRNAs (except for the H/ACA sRNA) were removed from the candidate pool, which reduce the number of candidates to 22 regions. The comparative analysis selected 106 regions within 95 IGRs. Based on the quality of the sequence alignments, we kept 65 of them for further analysis. Within the 73 candidates found by both approaches, 14 regions were common. Finally the comparison of these 73 regions using BlastN (W = 7) against the *P*. *abyssi *genome itself allowed the identification of nine additional regions corresponding to genomic repeats. Click here for file

Additional file 2 **Table S1**: List of the 82 predicted regions from the combined computational screens. Click here for file

Additional file 3 **Figure S2**: Primer extension experiments on total RNAs extracted from cells in entry in stationary phase. Length marker (M) corresponds to the sequence (T) of sRkB locus amplified from oligo sRkB_F (Additional file 7, Table S2) with the Thermo sequenase cycle sequencing kit from USB. (A) Primers matching pre-Cr and crRNAs. Reverse transcription arrests are denoted by small arrows. Direct repeats of CRISPR loci are highlighted in grey. (B) Primers matching ncRNAs as indicated. Click here for file

Additional file 4 **Figure S3**: (A) Sequence alignment of sRk11, sRk28, sRk33 and sRk61 related loci within the thermococcal genomes. The 5' and 3' ends of ncRNAs are specified. (B) Sequence alignment of the six loci related to sRkB/sRkC loci within the *P*. *abyssi *genome. Capital and small letters are for intergenic and ORF, respectively. The consensus BRE/TATA box promoter sequences are underlined. The 5' (arrows) and 3' (brackets) ends from CR-RT-PCR products are indicated. Black nucleotides indicate conserved nucleotides within the transcribed regions and highlighted nucleotides specify sequence variations. Promoter sequences are underlined. Secondary structure features are symbolized on top of each sequences: <<>> for stems, (()) for pseudoknots and * for covariation or G->U/U->G mutations preserving base-pairing. Click here for file

Additional file 5 **Figure S4**: (A) Sequence alignment (as denoted in Additional file 4, Figure S3) of the three loci related to sRk49 locus found in the *P*. *abyssi *(sRk49, sR49.2 and sRk49.3) and *P*. *horikoshii *genomes. (B) Detection of sRk49 by Northern blotting. Source of RNAs, markers and sR26 control are as indicated in Figure 1B. (C) Gene maps drawn to scale with transcripts depicted in red. Click here for file

Additional file 6 **Figure S5**: Sequence alignment (as denoted in Additional file 4, Figure S3) of the sixteen sequences related to sRk48/sRk52 loci found in the *P*. *abyssi *(Pab)*, P*. *horikoshii *(Pho), *P*. *furiosus *(Pfu), *T*. *sibiricus *(Tsi) *and T*. *kodokarensis *(Tko) genomes. Click here for file

Additional file 7 **Table S2**: List of oligonucleotides used in this study. Click here for file
