# Supplementary material for: Identification of CRISPR and riboswitch related RNAs among novel noncoding RNAs of the euryarchaeon Pyrococcus abyssi
Source: BMC Genomics. 2011 Jun 13;12:312. doi: 10.1186/1471-2164-12-312 (PMC3124441; doi:10.1186/1471-2164-12-312)
Supplement: Additional file 1 — Figure S1: Strategy for ncRNA candidate predictions. An initial set of candidates resulted from two complementary prediction methods. The bias composition selected 73 regions within 67 IGRs. The predicted regions expressing known ncRNAs (except for the H/ACA sRNA) were removed from the candidate pool, which reduce the number of candidates to 22 regions. The comparative analysis selected 106 regions within 95 IGRs. Based on the quality of the sequence alignments, we kept 65 of them for further analysis. Within the 73 candidates found by both approaches, 14 regions were common. Finally the comparison of these 73 regions using BlastN (W = 7) against the P. abyssi genome itself allowed the identification of nine additional regions corresponding to genomic repeats. [file 1471-2164-12-312-S1.PDF]

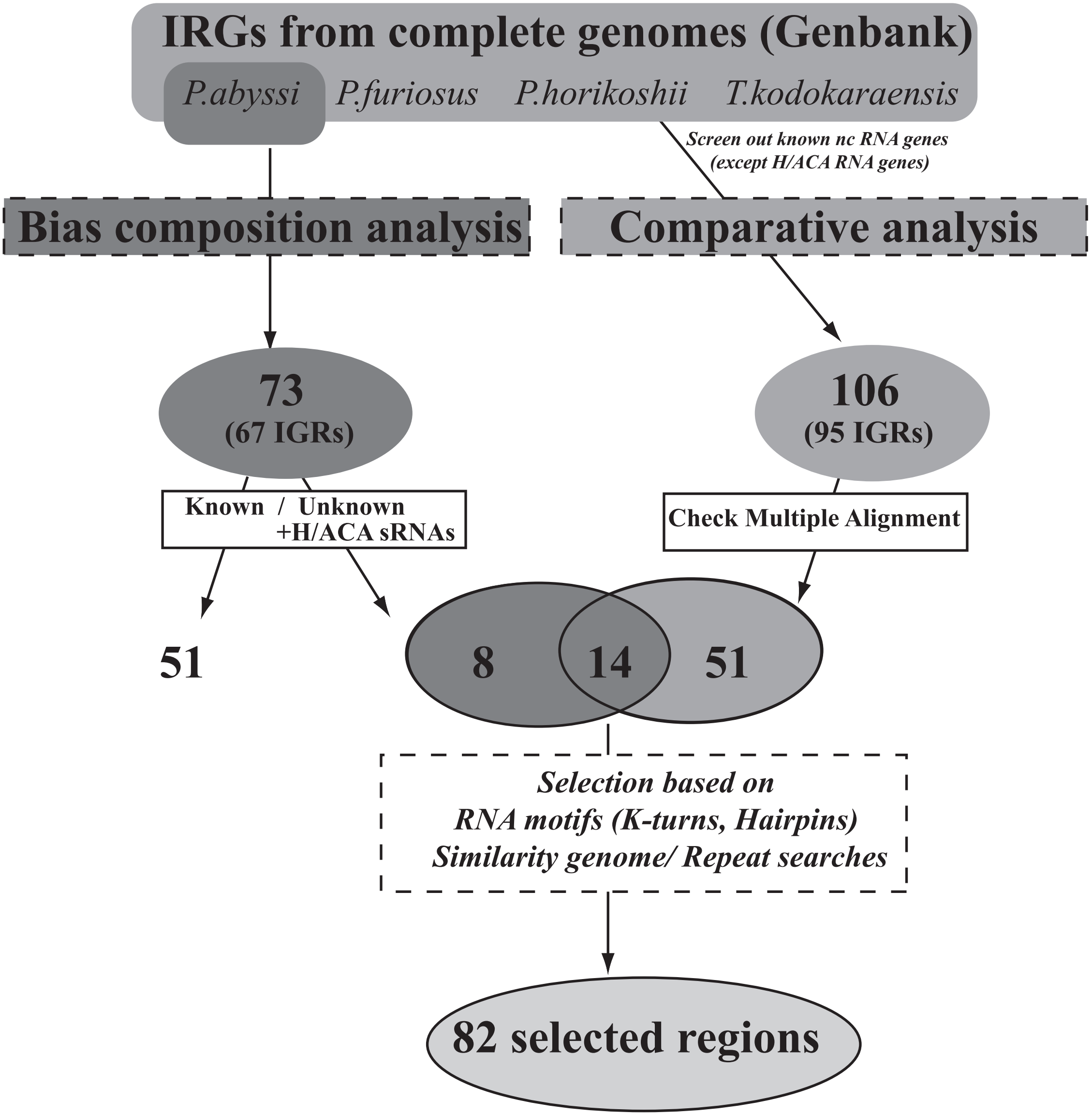
IRGs from complete genomes (Genbank)
P.abyssi
P.furiosus
P.horikoshii
T.kodokaraensis
Screen out known nc RNA genes
(except H/ACA RNA genes)
Bias composition analysis
Comparative analysis
73
(67 IGRs)
106
(95 IGRs)
Known / Unknown
+H/ACA sRNAs
Check Multiple Alignment
51
8
14
51
Selection based on
RNA motifs (K-turns, Hairpins)
Similarity genome/ Repeat searches
82 selected regions
