# Supplementary material for: Identification of CRISPR and riboswitch related RNAs among novel noncoding RNAs of the euryarchaeon Pyrococcus abyssi
Source: BMC Genomics. 2011 Jun 13;12:312. doi: 10.1186/1471-2164-12-312 (PMC3124441; doi:10.1186/1471-2164-12-312)
Supplement: Additional file 2 — Table S1: List of the 82 predicted regions from the combined computational screens. [file 1471-2164-12-312-S2.PDF]

Table S1

name	Validated	predicted start	predicted end	predicted lenght	Previously predicted/annotated
RNA1		53	124	71	
PfQ12y		5061	5187	126	PfQ12[1]
RNA2		16318	16461	143	
RNA3		49486	49576	90	
abi1		58858	58987	129	
RNA4		59344	59422	78	PfQ13[1]
RNA5		122730	123156	426	PfQ1[1]
RNA7	CRISPR1	148457	148597	140	CRISPR
RNA8	CRISPR1	149316	149538	222	CRISPR
RNA9		160602	160663	61	
RNA10		164355	164473	118	
RNA11	sRk11	197408	197477	69	
RNA12	Pab19[2]	204858	205038	180	Pab19[2]/
RNA16	Pab21[2]	230516	230634	118	Pab21[2]/sR9-Pf1[1,5]
RNA17		259798	259911	113	
RNA18	Pab160[2]	289023	289153	130	Pab160[2]/Pf9[1,5]
RNA19	HgcF[1],Pab35[2]	318187	318426	239	Pab35[2]/Pf6[1,5]
RNA20		337698	337835	137	
RNA21		355801	355883	82	
RNA22	HgcG[1], Pab40[2]	382342	382606	264	Pab40[2]/Pf7[1,5]
RNA23		383538	383602	64	
A		395121	395273	152	
RNA24		410819	410963	144	
RNA25		482452	482600	148	CRISPR
49.2		482980	483130	150	
RNA27		488912	488989	77	
RNA28	sRk28	527697	527833	136	
RNA29		543360	543434	74	
RNA31		574038	574187	149	
RNA33	sRk33, sscA[1]	636764	636919	155	PfQ11
RNA34		639639	639725	86	
RNA35		644754	644820	66	
RNA36		683491	683640	149	
RNA37		690024	690232	208	Pf5[1]
RNA38		784607	784688	81	
RNA39		790959	791021	62	
RNA40		798388	798529	141	
B	sRkB	809887	810256	369	
RNA41		834000	834132	132	
49.3		836510	836660	150	
RNA42		850691	850837	146	
RNA43		862333	862529	196	PfQ12[1]

RNA44		862631	862707	76	
Pab4		877493	877666	173	
RNA45		884120	884228	108	
RNA46		908833	908975	142	PfQ12[1]
RNA47		913309	913447	138	
RNA48	sRk48	985849	986002	153	PfQ12[1]
PfQ12b		1023891	1024016	125	PfQ12[1]
RNA49	sRk49	1067710	1067857	147	
RNA50	Pab91[2]	1074502	1074589	87	Pab91[2]/Pf4[1,5]
E		1079752	1080083	331	
abi2		1093067	1093124	57	
RNA51		1101361	1101414	53	
RNA52	sRk52	1104008	1104286	278	PfQ12[1]
RNA53		1119660	1119799	139	
RNA76		1125316	1125400	55	PfQ8[1]
D		1183102	1183233	131	
RNA54		1198159	1198249	90	
RNA55		1218183	1218247	64	
RNA56		1220201	1220350	149	PfQ6[1]
RNA57		1254313	1254409	96	
RNA58		1271361	1271472	111	
RNA59		1333783	1333858	75	
RNA60	hgcE[1], Pab105[2]	1335653	1335801	148	Pab105[2]/Pf3[1,5]
RNA61	sRk61	1348633	1348700	67	
F		1351205	1351361	156	
RNA62		1397186	1397290	104	
RNA63		1459510	1459626	116	
RNA64		1478385	1478529	144	
RNA65		1499982	1500129	147	
RNA66		1578824	1578902	78	
RNA67		1610197	1610273	76	
C	sRkC	1612986	1613347	361	
RNA68		1613567	1613716	149	
RNA69		1715287	1715350	63	
RNA70		1720605	1720715	110	PfQ2[1]
RNA71		1720769	1720821	52	
RNA72		1730555	1730636	81	
RNA73		1754421	1754563	142	
RNA74	CRISPR2	1760267	1760411	144	
RNA75	CRISPR2	1760842	1761020	178	

This study in red; [1] [22]; [2] [16]; [3][43]; [4] [60]; [5][35]
