# Supplementary figures and images for: Identification of CRISPR and riboswitch related RNAs among novel noncoding RNAs of the euryarchaeon Pyrococcus abyssi
Source: BMC Genomics. 2011 Jun 13;12:312. doi: 10.1186/1471-2164-12-312 (PMC3124441; doi:10.1186/1471-2164-12-312)

A

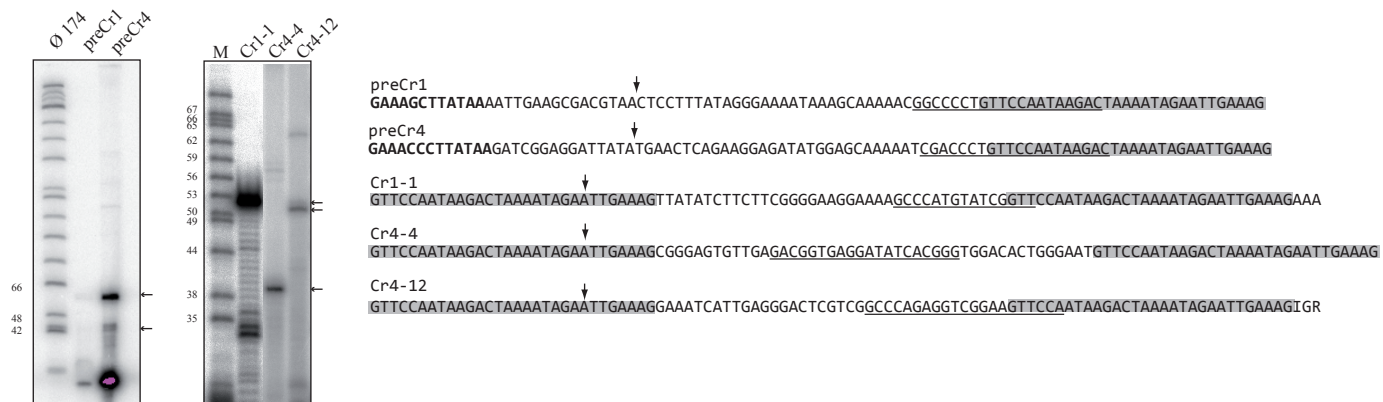

B

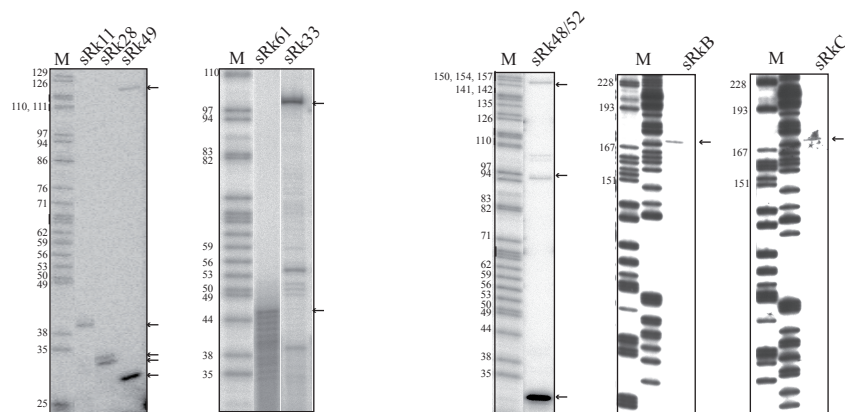

Supplement: Additional file 3 — Figure S2: Primer extension experiments on total RNAs extracted from cells in entry in stationary phase. Length marker (M) corresponds to the sequence (T) of sRkB locus amplified from oligo sRkB_F (Additional file 7, Table S2) with the Thermo sequenase cycle sequencing kit from USB. (A) Primers matching pre-Cr and crRNAs. Reverse transcription arrests are denoted by small arrows. Direct repeats of CRISPR loci are highlighted in grey. (B) Primers matching ncRNAs as indicated. [file 1471-2164-12-312-S3.PDF]
