# Supplementary material for: Identification of CRISPR and riboswitch related RNAs among novel noncoding RNAs of the euryarchaeon Pyrococcus abyssi
Source: BMC Genomics. 2011 Jun 13;12:312. doi: 10.1186/1471-2164-12-312 (PMC3124441; doi:10.1186/1471-2164-12-312)
Supplement: Additional file 4 — Figure S3: (A) Sequence alignment of sRk11, sRk28, sRk33 and sRk61 related loci within the thermococcal genomes. The 5' and 3' ends of ncRNAs are specified. (B) Sequence alignment of the six loci related to sRkB/sRkC loci within the P. abyssi genome. Capital and small letters are for intergenic and ORF, respectively. The consensus BRE/TATA box promoter sequences are underlined. The 5' (arrows) and 3' (brackets) ends from CR-RT-PCR products are indicated. Black nucleotides indicate conserved nucleotides within the transcribed regions and highlighted nucleotides specify sequence variations. Promoter sequences are underlined. Secondary structure features are symbolized on top of each sequences: <<>> for stems, (()) for pseudoknots and * for covariation or G->U/U->G mutations preserving base-pairing. [file 1471-2164-12-312-S4.PDF]

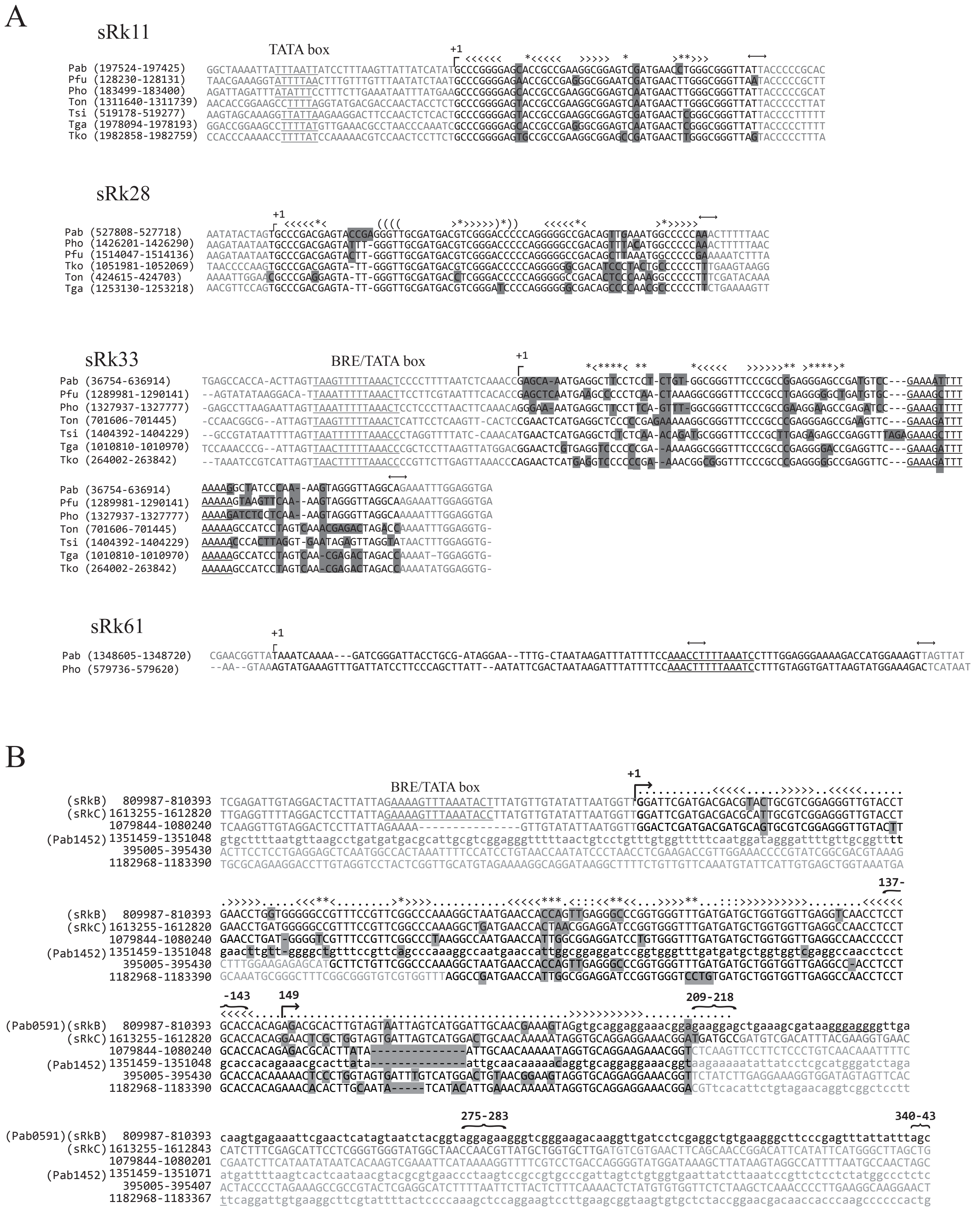
A
sRk11
TATA box
+1
Pab (197524-197425)
Pfu (128230-128131)
Pho (183499-183400)
Ton (1311640-1311739)
Tsi (519178-519277)
Tga (1978094-1978193)
Tko (1982858-1982759)
sRk28
Pab (527808-527718)
Pho (1426201-1426290)
Pfu (1514047-1514136)
Tko (1051981-1052069)
Ton (424615-424703)
Tga (1253130-1253218)
sRk33
BRE/TATA box
Pab (36754-636914)
Pfu (1289981-1290141)
Pho (1327937-1327777)
Ton (701606-701445)
Tsi (1404392-1404229)
Tga (1010810-1010970)
Tko (264002-263842)
sRk61
Pab (1348605-1348720)
Pho (579736-579620)
B
BRE/TATA box
(sRkB) 809987-810393
(sRkC) 1613255-1612820
1079844-1080240
(Pab1452) 1351459-1351048
395005-395430
1182968-1183390
137-
-143
149
209-218
(Pab0591)(sRkB) 809987-810393
275-283
340-43
(sRkC) 1613255-1612843
1079844-1080201
395005-395407
1182968-1183367
