# Supplementary material for: Identification of CRISPR and riboswitch related RNAs among novel noncoding RNAs of the euryarchaeon Pyrococcus abyssi
Source: BMC Genomics. 2011 Jun 13;12:312. doi: 10.1186/1471-2164-12-312 (PMC3124441; doi:10.1186/1471-2164-12-312)
Supplement: Additional file 5 — Figure S4: (A) Sequence alignment (as denoted in Additional file 4, Figure S3) of the three loci related to sRk49 locus found in the P. abyssi (sRk49, sR49.2 and sRk49.3) and P. horikoshii genomes. (B) Detection of sRk49 by Northern blotting. Source of RNAs, markers and sR26 control are as indicated in Figure 1B. (C) Gene maps drawn to scale with transcripts depicted in red. [file 1471-2164-12-312-S5.PDF]

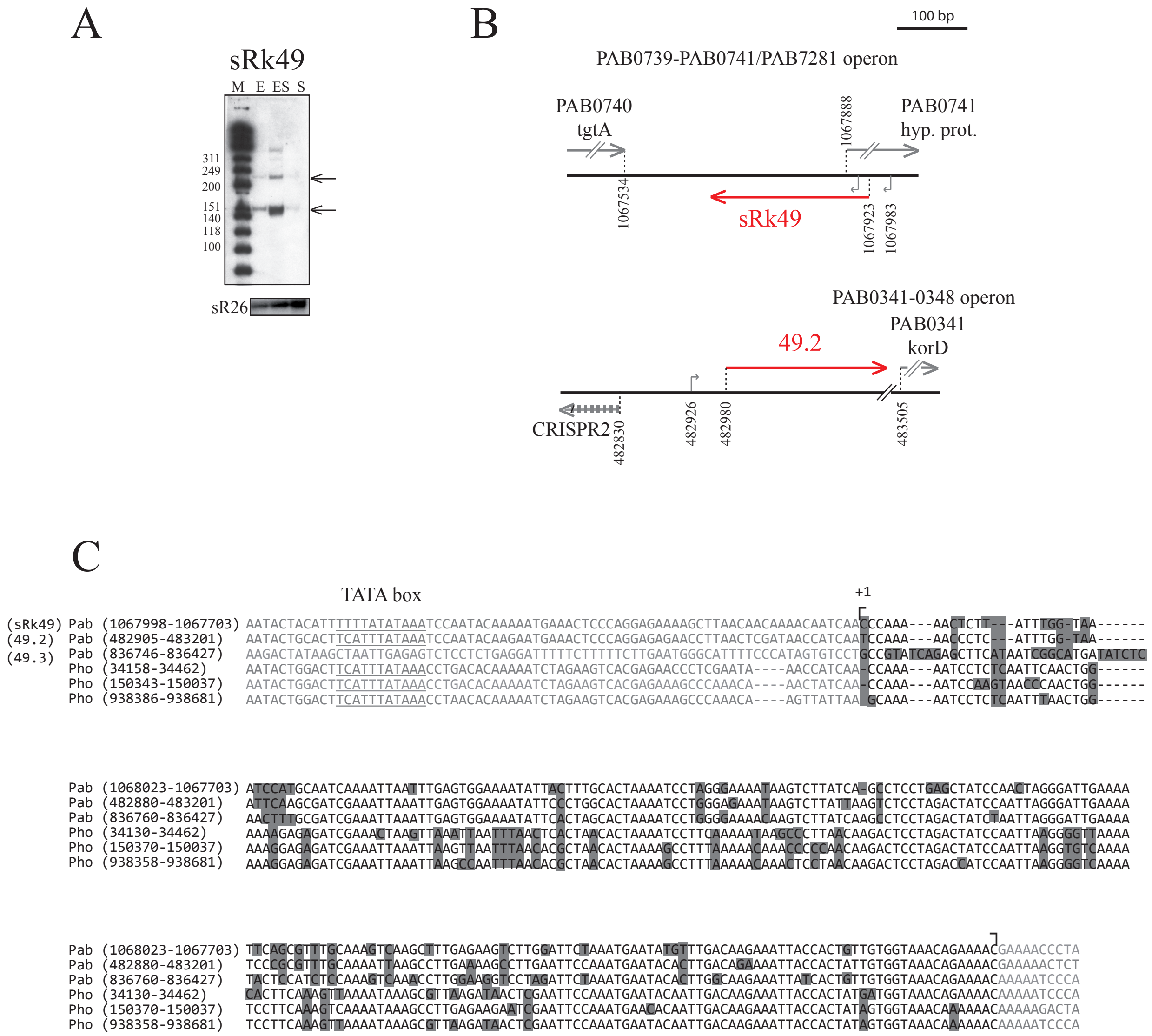
A
sRk49
M E ES S
311
249
200
151
140
118
100
sR26
B
100 bp
PAB0739-PAB0741/PAB7281 operon
PAB0740
tgtA
PAB0741
hyp. prot.
1067888
1067534
sRk49
1067923
1067983
PAB0341-0348 operon
PAB0341
korD
49.2
CRISPR2
482830
482926
482980
483505
C
TATA box
+1
(sRk49) Pab (1067998-1067703) AATACTACATTTTTTATATAAATCCAATACAAAAATGAAACTCCCAGGAGAAAAGCTTAACAACAAAACAATCAACCCAAA---AACTCTT---ATTTGG-TAA------
(49.2) Pab (482905-483201) AATACTGCACTTCATTTATAAATCCAATACAAGAATGAAACTCCCAGGAGAGAACCTTAACTCGATAACCATCAATCCAAA---AACCCTC---ATTTGG-TAA------
(49.3) Pab (836746-836427) AAGACTATAAGCTAATTGAGAGTCTCCTCTGAGGATTTTTCTTTTTCTTGAATGGGCATTTTCCCATAGTGTCCTGCCGTATCAGAGCTTCATAATCGGCATGATATCTC
Pho (34158-34462) AATACTGGACTTCATTTATAAACCTGACACAAAAATCTAGAAGTCACGAGAACCCTCGAATA----AACCATCAA-CCAAA---AATCCTCTCAATTCAACTGG------
Pho (150343-150037) AATACTGGACTTCATTTATAAACCTGACACAAAAATCTAGAAGTCACGAGAAAGCCCAAACA----AACTATCAA-CCAAA---AATCCAAGTAACCCAACTGG------
Pho (938386-938681) AATACTGGACTTCATTTATAAACCTAACACAAAAATCTAGAAGTCACGAGAAAGCCCAAACA----AGTTATTAA GCAAA---AATCCTCTCAATTTAACTGG------
Pab (1068023-1067703) ATCCATGCAATCAAAATTAATTTGAGTGGAAAATATTACTTTGCACTAAAATCCTAGGGAAAATAAGTCTTATCA-GCCTCCTGAGCTATCCAACTAGGGATTGAAAA
Pab (482880-483201) ATTCAAGCGATCGAAATTAAATTGAGTGGAAAATATTCCCTGGCACTAAAATCCTGGGAGAAATAAGTCTTATTAAGTCTCCTAGACTATCCAATTAGGGATTGAAAA
Pab (836760-836427) AACTTTGCGATCGAAATTAAATTGAGTGGAAAATATTCACTAGCACTAAAATCCTGGGGAAAACAAGTCTTATCAAGCCTCCTAGACTATCTAATTAGGGATTGAAAA
Pho (34130-34462) AAAAGAGAGATCGAAACTAAGTTAAATTAATTTAACTCACTAACACTAAAATCCTTCAAAAATAAGCCCTTAACAAGACTCCTAGACTATCCAATTAAGGGGTTAAAA
Pho (150370-150037) AAAGGAGAGATCGAAATTAAATTAAGTTAATTTAACACGCTAACACTAAAAGCCTTTAAAAACAAACCCCCAACAAGACTCCTAGACTATCCAATTAAGGTGTCAAAA
Pho (938358-938681) AAAGGAGAGATCGAAATTAAATTAAGCCAATTTAACACGCTAACACTAAAAGCCTTTAAAAACAAACTCCTAACAAGACTCCTAGACCATCCAATTAAGGGGTCAAAA
Pab (1068023-1067703) TTCAGCGTTTGCAAAGTCAAGCTTTGAGAAGTCTTGGATTCTAAATGAATATGTTTGACAAGAAATTACCACTGTTGTGGTAAACAGAAAACGAAAACCCTA
Pab (482880-483201) TCCCGCGTTTGCAAAATTAAGCCTTGAAAAGCCTTGAATTCCAAATGAATACACTTGACAGAAAATTACCACTATTGTGGTAAACAGAAAACGAAAAACTCT
Pab (836760-836427) TACTCCATCTCCAAAGTCAAACCTTGGAAGGTCCTAGATTCTAAATGAATACACTTGGCAAGAAATTATCACTGTTGTGGTAAACAGAAAACAAAAATCCCA
Pho (34130-34462) CACTTCAAAGTTAAAATAAAGCGTTAAGATAACTCGAATTCCAAATGAATACAATTGACAAGAAATTACCACTATGATGGTAAACAGAAAACAAAAATCCCA
Pho (150370-150037) TCCTTCAAAGTCAAAATAAAGCCTTGAGAAGAATCGAATTCCAAATGAACACAATTGACAAGAAATTACCACTATAGTGGTAAACAAAAAACAAAAAGACTA
Pho (938358-938681) TCCTTCAAAGTTAAAATAAAGCGTTAAGATAACTCGAATTCCAAATGAATACAATTGACAAGAAATTACCACTATAGTGGTAAACAAAAAACAAAAATCCCA
