# Supplementary material for: Identification of CRISPR and riboswitch related RNAs among novel noncoding RNAs of the euryarchaeon Pyrococcus abyssi
Source: BMC Genomics. 2011 Jun 13;12:312. doi: 10.1186/1471-2164-12-312 (PMC3124441; doi:10.1186/1471-2164-12-312)
Supplement: Additional file 6 — Figure S5: Sequence alignment (as denoted in Additional file 4, Figure S3) of the sixteen sequences related to sRk48/sRk52 loci found in the P. abyssi (Pab), P. horikoshii (Pho), P. furiosus (Pfu), T. sibiricus (Tsi) and T. kodokarensis (Tko) genomes. [file 1471-2164-12-312-S6.PDF]

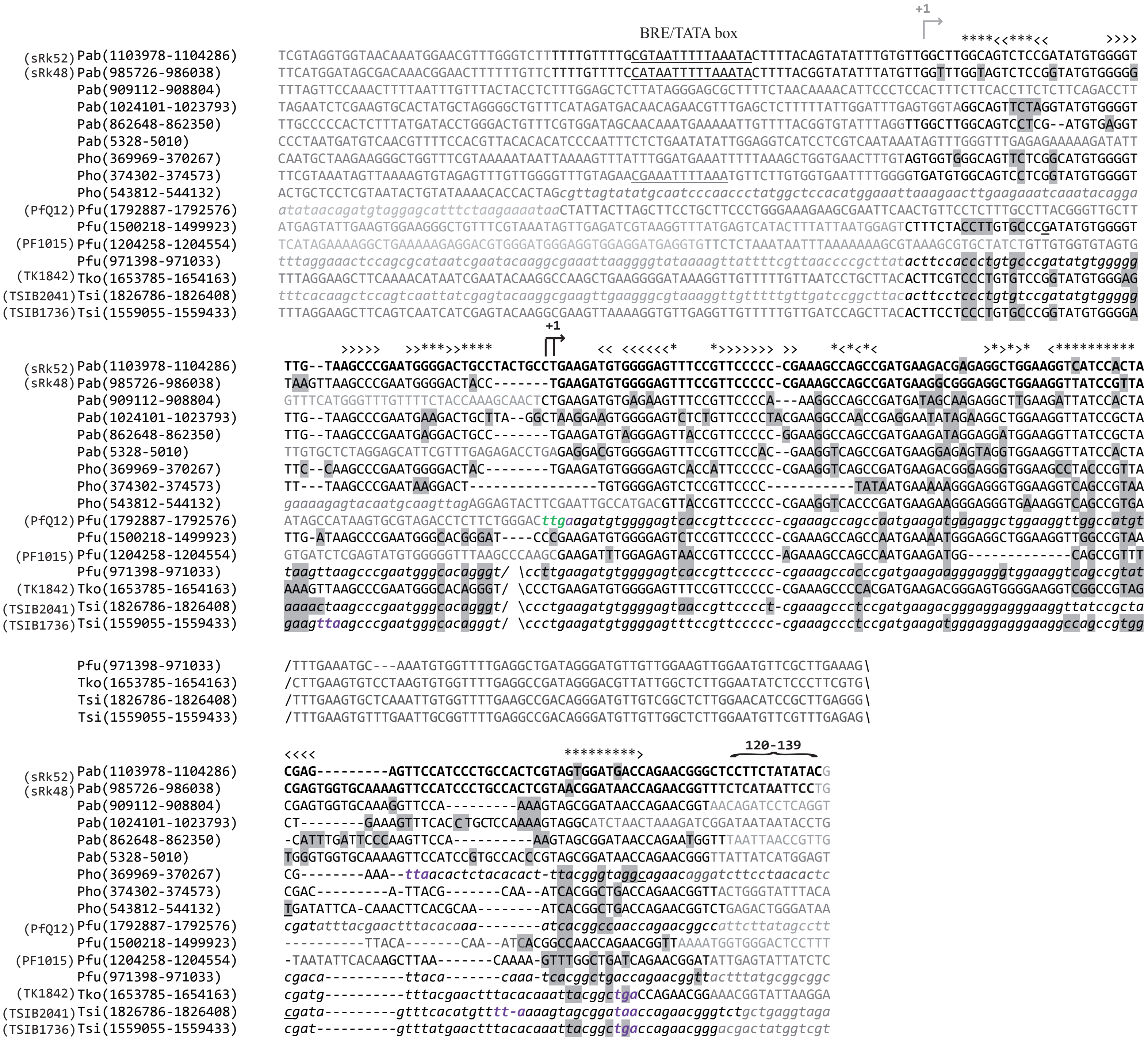
BRE/TATA box
+1
120-139
(sRk52) Pab(1103978-1104286)
(sRk48) Pab(985726-986038)
Pab(909112-908804)
Pab(1024101-1023793)
Pab(862648-862350)
Pab(5328-5010)
Pho(369969-370267)
Pho(374302-374573)
Pho(543812-544132)
(PfQ12) Pfu(1792887-1792576)
Pfu(1500218-1499923)
(PF1015) Pfu(1204258-1204554)
Pfu(971398-971033)
(TK1842) Tko(1653785-1654163)
(TSIB2041) Tsi(1826786-1826408)
(TSIB1736) Tsi(1559055-1559433)
