# Supplementary material for: Identification of CRISPR and riboswitch related RNAs among novel noncoding RNAs of the euryarchaeon Pyrococcus abyssi
Source: BMC Genomics. 2011 Jun 13;12:312. doi: 10.1186/1471-2164-12-312 (PMC3124441; doi:10.1186/1471-2164-12-312)
Supplement: Additional file 7 — Table S2: List of oligonucleotides used in this study. [file 1471-2164-12-312-S7.PDF]

Supplementary Table 2 : Primers used in this study

Name	Sequence (5′ to 3′)	Features
Cr1-1_F	GCCCATGTATCGGTTCCAA	NB on CRISPR1 spacer 1
Cr1-1_R	TTGGAACCGATACATGGGC	NB and RT on CRISPR1 spacer 1
Cr1-22_R	TGCACTCGCCGTTGCGAGCT	RT on CRISPR1 spacer 22
Pre-Cr1	GTCTTATTGGAACAGGGGCC	NB on CRISPR1 RNA precursor
Cr4-4_F	TGGAACTTCCGACCTCTGGGC	NB and RT on CRISPR4 spacer 4
Cr4-4_R	GCCCAGAGGTCGGAAGTTCCA	NB on CRISPR4 spacer 4
Cr4-12_F	CCCGTGATATCCTCACCGTC	NB and RT on CRISPR4 spacer 12
Cr4-12_R	GACGGTGAGGATATCACGGG	NB on CRISPR4 spacer 12
Cr4-28_F	GAAGGTCGCGGGTTCAAATC	NB on CRISPR4 spacer 28
Pre-Cr4	GTCTTATTGGAACAGGGCGT	RT on CRISPR4 RNA precursor
Cr2-2_F	GCAGGGCTAAGTTGGGCAAG	NB on CRISPR2 spacer 2
Cr2-2_R	CTTGCCCAACTTAGCCCTGC	NB on CRISPR2 spacer 2
Cr3-2-F	GTTCAATCTGTAGGAAATGC	NB on CRISPR3 spacer 2
Cr3-2-R	GCATTTCCTACAGATTGAAC	NB on CRISPR3 spacer 2
sRk11_F	GGCGGAGTCGATGAACCTGG	NB on sRk11 locus
sRk11_R	CCAGGTTCATCGACTCCGCC	NB and RT on sRk11 locus
sRk28_F	CGAGGGTTGCGATGACGT	NB on sRk28 locus
sRk28_R	ACGTCATCGCAACCCTCG	NB and RT on sRk28 locus
sRk33_F	GAAACCCGCCACAGAGGAGG	NB and RT on sRk33 locus
sRk33_R	CCTCCTCTGTGGCGGGTTTC	NB on sRk33 locus
sRk49_F	CCTGAGCTATCCAACTAGGG	NB on sRk49 locus
sRk49_R	CCCTAGTTGGATAGCTCAGG	NB and RT on sRk49 locus
sRk61_F	CTTATTAGCAAATTCCTATCGC	NB and RT on sRk61 locus
sRk61_R	GCGATAGGAATTTGCTAATAAG	NB on sRk61 locus
sRK48/52_F	GGGATGGAACTTTTGCACCA	NB and RT on sRk48/52 loci
sRK48/52_R	GGCCAGCCGATGAAGATAGG	NB on sRk48/52 loci
sRk48/52.1	GGATAATCTTCAAGCCTCTTGCT	NB on repetitive locus of sRk48/52 (909112-908804)
sRk48/52.2	GGATAACCTTCCAGCCTTCTATA	NB on repetitive locus of sRk48/52 (1024101-1023785)
sRk48/52.3	GATAACCTTCCATCCTCCTATCT	NB on repetitive locus of sRk48/52 (862648-862350)
sRk48/52.4	GGATAACCTTCCACCTACTCTCC	NB on repetitive locus of sRk48/52 (5328-5010)
RACEsRk48/52_r	GAACGGAAACTCCCCACATC	C-RACE on sRk48 and sRk52 loci
RACEsRk48/52_p	CCCCCGAAAGCCAGCCGATG	
sRkB_F	CAGCTCCTTCTCCGTTTCCT	NB and RT on sRkB locus
sRkB_R	AGGAAACGGAGAAGGAGCTG	NB on sRkB locus
sRkC_F	AGCATCATCAAACCCACCGG	NB on sRkB locus
sRkC-R	GGTGGTTGAGGCCAACCTCC	NB and RT on sRkB locus

sRkB/C.1_F	AGAATACATAACAAAAGTATT	NB on repetitive locus of sRkB/C (1079844-1080240)
sRkB/C.1_R	GCAATTATAAGTGCGTTTCTGT	NB on repetitive locus of sRkB/C (1079844-1080240)
sRkB/C.2_F	GAAAAATATTATCCTCGCATGG	NB on repetitive locus of sRkB/C (1351459-1351048)
sRkB/C.2_R	CCATGCGAGGATAATATTTTTC	NB on repetitive locus of sRkB/C (1351459-1351048)
sRkB/C.3_F	AACCGTTTCCTCCTGCACCT	NB on repetitive locus of sRkB/C (395005-395430)
sRkB/C.3_R	AGGTGCAGGAGGAAACGGTT	NB on repetitive locus of sRkB/C (395005-395430)
sRkB/C.4_F	CAGGACCCACCGGATCCTC	NB on repetitive locus of sRkB/C (1182968-1183390)
sRkB/C.4_R	GAGGATCCGGTGGGTCCTG	NB on repetitive locus of sRkB/C (1182968-1183390)
RACEsRkB_r	ATGACTAATTACTACAAGTGCGTCT	C-RACE on sRkB locus (R1)
RACEsRkB_p	AAAGCGATAAGGGAGGGGTT	
RACEsRkC_r	CAGGACCCACCGGATCCTC	C-RACE on sRkC locus (R2)
RACEsRkC_p	GGACTGCAACAAAAATAGGTGCAGG	
RACEsRkC_r	GAACTCGCTGGTAGTGATTAGT	C-RACE on sRkC locus (R3)
RACEsRkC_p	GGACTGCAACAAAAATAGGTGCAGG	
OLT7B216	**CCGGGAATTCTAATACGACTCACTATA**GGATTCGATGACGACGTACTG	PCR oligo product as template for sRkB216 RNA
OL3'B216	GCTCCTTCTCCGTTTCCTCCTG	
OLT7B3	**CCGGGAATTCTAATACGACTCACTATA**GGCTAATGAACCACCAGTTGA	PCR product as template for sRkB*
3mutKT_CCA	ATCAAACCCACCGGGCCCGCAACTGGTG	
5mutKT_CCA	GGGCCCGGTGGGTTTGATGCAGCTGGTG	
OL3'B216	GCTCCTTCTCCGTTTCCTCCTG	
OLT7C211	**CCGGGAATTCTAATACGACTCACTATA**GGTTGGATTCGATGACGACG	PCR product as template for sRkC211 RNA
OL3'C211	TCATCCGTTTCCTCCTGCACC	

sRkX_F or R (X : locus name) : complementary to the forward (F) strand or reverse (R)
NB : primer used for Northern blotting analysis
RT : primer used for mapping 5'end of transcripts of each locus
RACEsRkXr or p (X : locus name) : flanked primers used for mapping 5' and 3' ends by C-RACE analysis
